# Defining Remoteness from Health Care: Integrated Research on Accessing Emergency Maternal Care in Indonesia

**DOI:** 10.3934/publichealth.2015.3.257

**Published:** 2015-07-01

**Authors:** Bronwyn A Myers, Rohan P Fisher, Nelson Nelson, Suzanne Belton

**Affiliations:** 1 Research Institute for the Environment and Livelihoods, Charles Darwin University, Darwin, Northern Territory 0909, Australia; 2 Department of Health, South Central Timor District, So'E, Matarak, East Nusa Tenggara Province, Indonesia; 3 Menzies School of Health Research, Charles Darwin University, Casuarina, Northern Territory 0820, Australia

**Keywords:** maternal health care, access, remoteness, eastern Indonesia, Geographic Information Systems

## Abstract

The causes of maternal death are well known, and are largely preventable if skilled health care is received promptly. Complex interactions between geographic and socio-cultural factors affect access to, and remoteness from, health care but research on this topic rarely integrates spatial and social sciences. In this study, modeling of travel time was integrated with social science research to refine our understanding of remoteness from health care. Travel time to health facilities offering emergency obstetric care (EmOC) and population distribution were modelled for a district in eastern Indonesia. As an index of remoteness, the proportion of the population more than two hours estimated travel time from EmOC was calculated. For the best case scenario (transport by ambulance in the dry season), modelling estimated more than 10,000 fertile aged women were more than two hours from EmOC. Maternal mortality ratios were positively correlated with the remoteness index, however there was considerable variation around this relationship. In a companion study, ethnographic research in a subdistrict with relatively good access to health care and high maternal mortality identified factors influencing access to EmOC, including some that had not been incorporated into the travel time model. Ethnographic research provided information about actual travel involved in requesting and reaching EmOC. Modeled travel time could be improved by incorporating time to deliver request for care. Further integration of social and spatial methods and the development of more dynamic travel time models are needed to develop programs and policies to address these multiple factors to improve maternal health outcomes.

## 1. Introduction

Almost all (99%) maternal deaths occur in developing countries [1], and addressing this inequity is one of the Millennium Development Goals. Most maternal deaths are caused by bleeding, infection, high blood pressure, unsafe abortion or obstructed labour: all conditions that need not be fatal if timely emergency obstetric care (EmOC) is received. There are many potential barriers to timely access to EmOC and these delays have been conceptualised in the “three delays model” of Thaddeus and Maine: delays in (i) making the decision to seek care; (ii) reaching a health facility; and (iii) receiving appropriate health care (Table 1) [2]. The second delay comprises the five dimensions of access identified and defined by Penchansky and Thomas [3]: availability, accessibility, accommodation, affordability and acceptability. The second delay is strongly influenced by geographic factors [4] and Geographic Information System (GIS) tools are increasingly being used to measure accessibility of health facilities by modelling travel time, taking into account the effects of topography, available transport and seasonal barriers [5]–[10]. Whilst providing measures that can be quantified, mapped, and easily incorporated into development planning activities, these studies fail to address all the issues that affect access to health services particularly the complex chain of decisions required for initially seeking care and then obtaining transport.

**Table 1.** The sequence of potential delays to accessing maternal health care.

	Dimensions influencing access to maternal health care	Dominant factors
First delay	Recognition of need for care	Sociocultural
Second delay	Acceptability of health care	Sociocultural
	Adequacy of health care	
	Affordability of health care	Financial
	Accessibility of health care	Geographical
	Availability of health care	
	Location of target population	
Third delay	Effectiveness of care	Clinical skills & resources

Definitions of “remoteness” generally refer to distance or travel time from services [11]. In the case of maternal emergencies, it is recommended that basic EmOC be accessible within two hours [12] and so we measured remoteness as the proportion of the population more than two hours modelled travel time from EmOC. It is clear from the three delays model and decades of associated research that access to EmOC is not solely determined by travel time but is influenced by a complex array of socio-cultural, financial and geographic factors [13],[14]. There is a growing acceptance of the benefits of integrating spatial and social sciences in order to improve our understanding of such complex problems. As stated by Fielding & Cisneros-Puebla [15] (p. 353), “from geography, social science gains better sensitivity to scale, place, context, and flows. From social science, geography can gain better practices for documenting processes and cultural variation, systematic code-based data management, and formal analytical strategies”. However, investigations of access to health services rarely combine geographic and socio-cultural research techniques.

This paper explores the concept of remoteness from EmOC in a developing country context in terms of geographic access and other factors that limit access to EmOC services. Ethnographic research by Belton et al. [16] is a companion study to this paper, and describes the many factors influencing access to EmOC for cases of maternal deaths in one subdistrict in eastern Indonesia. Although inequities in access to health services are known, causes of these inequities are less known, particularly for groups without ready access [17],[18]. The ethnographic research [16] showed, for some cases, the delay in deciding to seek care was related to the stigma of unmarried pregnancy and resulted in death close to EmOC. However, for many cases attempts were made to seek help and travel time had multiple influences on the delays in seeking and reaching care: the time to deliver the request for assistance, for a midwife or ambulance to arrive, and/or for the woman to travel to health care [16].

## 2. Study location

**Figure 1.**
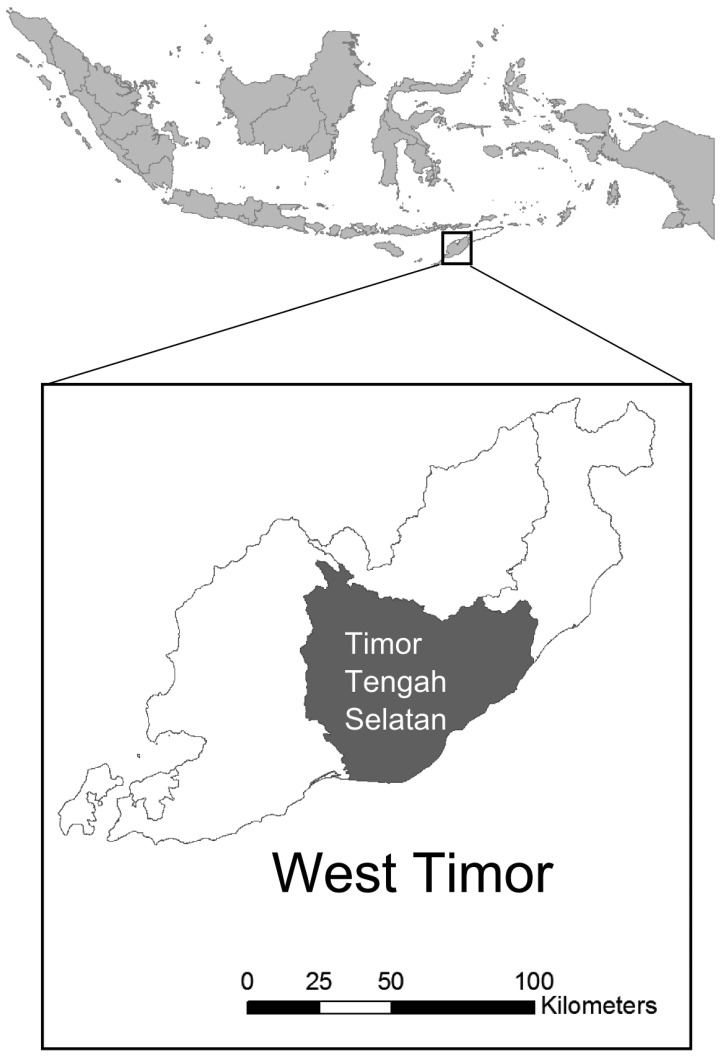
Study district of South Central Timor (TTS) in West Timor in eastern Indonesia.

This study investigated one district, South Central Timor (TTS), in the eastern Indonesia province of East Nusa Tenggara (NTT) (Figure 1). NTT is one of the poorest provinces in Indonesia and, in 2010, had higher maternal mortality rates than those for Indonesia as a whole. In NTT province there are approximately one million fertile-aged women (15–45 years) which is 43% of the female population [19]. In 2010, the district of TTS had more than double the national rate of maternal mortality and a relatively low proportion of births were attended by trained assistants (Table 2). Maternal deaths are underreported: e.g. in our ethnographic study [16], we encountered two unreported cases of maternal death without seeking unreported cases.

The landscape of TTS is largely rugged and there is a short and intense wet season. Marginal subsistence farming is the dominant livelihood, with many communities experiencing periods of hunger every year. The provision of services to the rural population is difficult because roads are few, generally of poor quality, and frequently impassable in the wet season due to flooding or landslides. For many people, accessing health services requires walking to the nearest road and the use of public transport where available. It is not uncommon for people in need of emergency care to be carried for up to five kilometers to a location that can be reached by motorised transport.

**Table 2.** Population, maternal mortality ratio (MMR; maternal deaths per 100,000 live births) and attendance at birth by trained assistant in 2010 (data from National, Provincial and District health reports).

	Population	MMR	Births with trained assistance (%)
	(x 1,000)	(/100,000 live births)	
Indonesia	237,641	208	82
NTT province	4,449	271	76
TTS district	441	596	72

Health services in TTS include one hospital in the district capital (So'E) and clinics in each of the 32 sub-districts. In 2013, there were four clinics that provided basic EmOC and one hospital that provided comprehensive EmOC in TTS. These five EmOC facilities cater for 420,000 people which is consistent with the recommended ratio of EmOC facilities per head of population [12]. Each sub-district clinic manages a variety of health facilities at the village level, including village maternity posts (*polindes*), integrated service posts for children under five years old (*posyandu balita*) and mobile clinics (*pusling*). However in 2010, only 59% of pregnant women attended the four recommended antenatal visits [20]. Each village midwife is responsible for a village maternity post however is not required to live in the village. In 2010 less than half the villages in TTS had a resident midwife [21].

## 3. Methods

### 3.1. Outline of methods used

Travel time to EmOC was modelled using four travel scenarios. In addition population distribution was modelled for each sub-district in TTS. For maternal emergency, it is recommended that basic emergency obstetric care (EmOC) be accessible within two hours [12] and so the index of remoteness was the proportion of the population more than two hours from EmOC. Mean maternal mortality ratio (numbers of maternal deaths per 100,000 live births) for a five year period was plotted against this remoteness index for each sub-district. One sub-district with a relatively high maternal mortality ratio and a relatively low remoteness index was selected for an ethnographic study of cases of maternal deaths [16]. For this selected sub-district, geographic access to EmOC was investigated with greater spatial resolution, and related to the circumstances surrounding the maternal deaths reported in the ethnographic study. The sources of these data are listed in Table 3. Lack of data and local expertise are often limitations for this type of analysis in developing countries [22]. In this study we used readily available free data and software and worked with local health staff to carry out the analysis. Author N, a district health department officer, plotted population distribution, assisted in conducting the travel time analysis and provided local knowledge used to validate modeling of flood prone locations.

**Table 3.** The sources of data used for each layer of the model of travel time in this study.

Data Source	Derived Model Layer
Landsat Imagery	Vegetation
SRTM DEM	Slope, stream networks
Local Government Survey (2010)	Roads, administrative boundaries
Topographic maps (1996)	Population distribution district level
Google Earth	Population distribution sub-district level

### 3.2. Estimations of travel time to Emergency Obstetric Care (EmOC) for TTS district

Travel time was modelled using a raster based cost-distance analysis approach. Whilst travel time modelling in the developed world is usually conducted using network analysis techniques based on transport infrastructure (road and rail networks) in regions without extensive transport infrastructure raster analysis is more useful as it explicitly calculates travel time for all locations in the modelled region [23],[24]. This approach is thus more commonly used in developing countries [25] with remote rural populations often distant from transport infrastructure such as in the case study region considered in this paper.

Cost distance analysis is based on a “cost” grid where each cell is assigned a “time –cost” for travelling through that cell. The time cost for each cell is determined by the land cover or transport infrastructure type in that cell. The cost distance analysis function then calculates the travel time for every cell in a grid to a defined destination point. Cost distance analysis is a standard raster GIS analysis function. For this study AccessMOD©, a plugin for ArcView 3 [8], was used to facilitate the cost distance analysis as the plugin provided a simplified method for producing the input “cost” and resulting Travel Time grids and ArcView 3 was the most commonly available GIS application available to our local collaborators. These two factors aided local collaboration and capacity building through the research process. Whilst ArcView 3 is not current software the cost distance analysis technique used is the same as employed by newer GIS packages.

To calculate travel time, vegetation, road, and water course data were combined to produce a raster layer with each grid cell allocated a travel time value based on an estimated average speed of transit. A vegetation cover classification was derived from Landsat imagery with four classes (forest, scrub, rocky and grassland); national road data, updated a year prior to the study, were obtained from the district government; and a stream network was created using digital elevation data (STRM) and hydrological analysis tools. The road and stream network data were rasterized overlain on the land cover data and resampled to a 50 meter grid resolution. A travel speed was allocated to each land-cover type based on four travel time scenarios: two transport scenarios in wet and dry seasons. The two transport scenarios were: (1) travel by clinic ambulance (“car”) on all roads and walking elsewhere, and (2) public transport (“bus”) on major roads with travel elsewhere by walking. Ambulances can travel on minor roads whereas buses are only available on provincial and national roads. So for the bus scenario, local roads were attributed with walking speed. Streams with a high network order (Strahler index > 6) were included as barriers to travel in the estimation of travel time. The speeds for each mode of travel (Table 4) were based on local knowledge. The travel time grids were intersected with health facility locations to calculate travel time to EmOC. The resultant grids were then reclassified into five zones of travel time to EmOC: 0–30, 30–60, 60–90, 90–120, and >120 minutes.

**Table 4.** Travel speed attributed to land-cover and road types for travel scenarios of road travel by ambulance (“car”) or public transport (“bus”) and walking elsewhere, in the dry or wet season.

Class	Land cover	Travel speed (km/h)			
		Wet season		Dry Season	
		Car	Bus	Car	Bus
0	Grass	3	3	4	4
1	Forest	2	2	3.5	3.5
2	Scrub	2	2	3.5	3.5
3	Rocky	1	1	2.5	2.5
4	Local Road	10	3.5	15	4
5	Provincial Road	25	10	30	10
6	National Road	25	40	60	40

### 3.3. Population distribution

**Figure 2.**
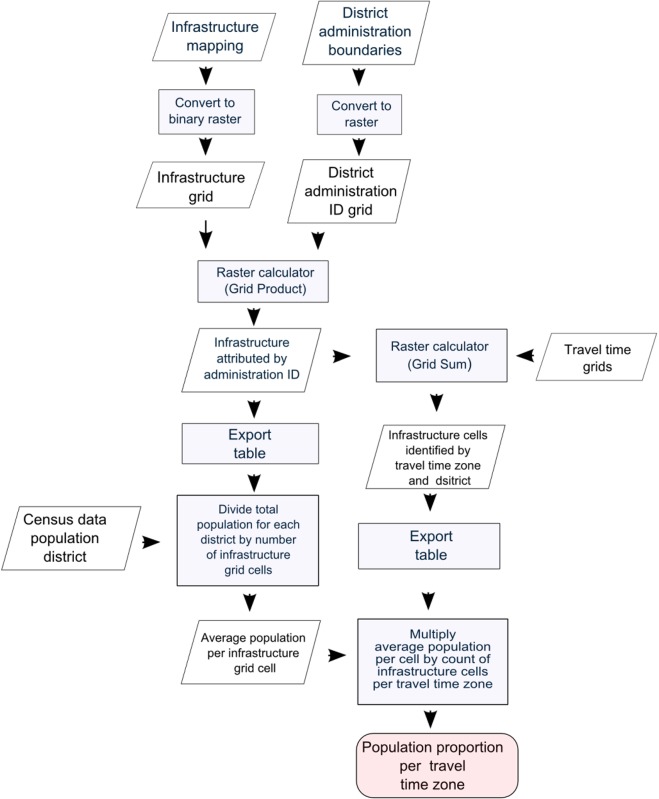
Processing flowchart for assessing remoteness from emergency obstetric care in Timor Tengah Selatan district.

To assess the proportion of the population more than two hours estimated travel time from EmOC (i.e. the remoteness index) a population distribution grid was derived from a map of built infrastructure produced in 1996 but there had been little development of this area since that time. The infrastructure mapping was converted to grid data and merged with sub-district boundary data for which recent population census figures were available [20]. The average number of people per infrastructure cell was then calculated by dividing the total population by the number of infrastructure grid cells for each district. This population distribution data were then combined with the travel time grids to produce the proportion of the population remote from EmOC. These steps are summarised in Figure 2.

### 3.4. Correlation between maternal mortality ratio and remoteness index

Maternal mortality ratio for each sub-district was calculated as the number of maternal deaths per 100,000 live births for each sub-district for each year for 2008 to 2012 (inclusive). The mean values for this five year period were calculated and plotted against the remoteness index for the wet and dry season. Maternal mortality ratio was modelled as a function of remoteness index and season (dry season = 1; wet season = 0) using a generalised linear model with a Poisson distribution and a log link. In order to compare the magnitude of effects between binary (e.g. season) and continuous (e.g. remoteness index) covariates, the remoteness index was standardized by two standard deviations [26].

### 3.5. Estimations of travel time for the study sub-district

**Figure 3.**
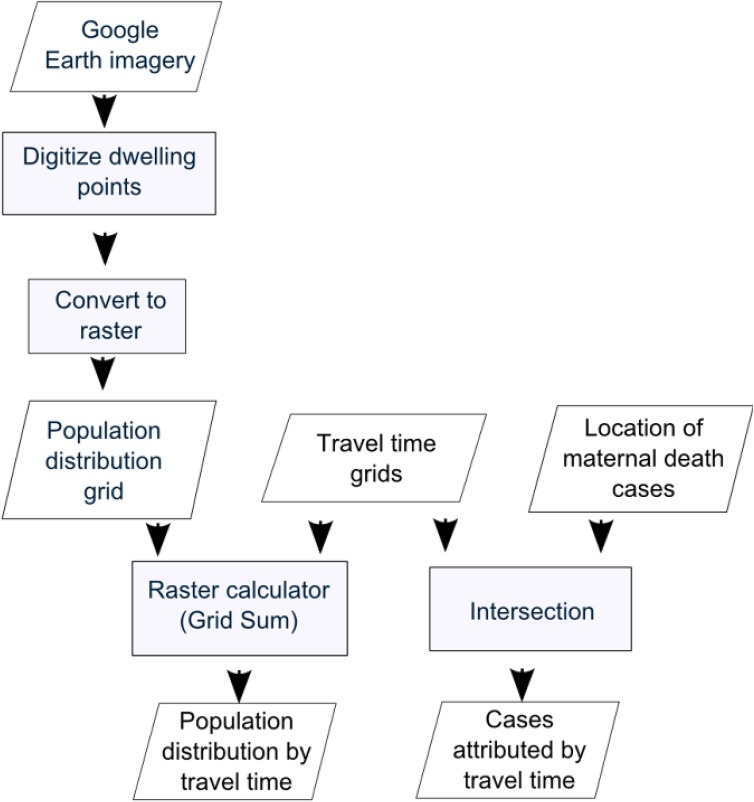
Processing flow chart for determining remoteness from emergency obstetric care in a selected sub-district.

A sub-district with relatively high maternal mortality ratio and relatively low remoteness index was selected for an ethnographic study of cases of maternal death (described in [16]). This selection deliberately targeted extreme cases as they often reveal more information through activating more actors and more basic mechanisms in the situation studied [27]. A higher resolution and more up-to-date population distribution layer was created for this sub-district by mapping dwellings manually using Google Earth imagery. A total of 1598 points were exported into a GIS format and then combined with the travel time grids as shown in Figure 3. The locations of the maternal deaths that were the subject of the interviews were also identified and compared to the travel time grids.

## 4. Results

**Figure 4.**
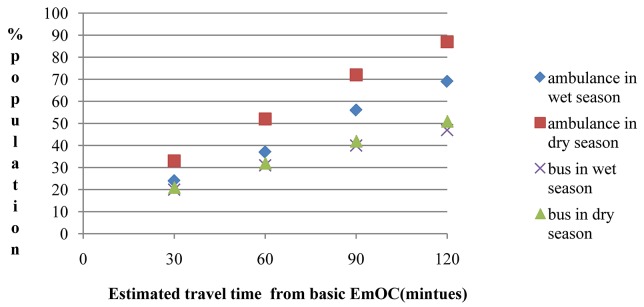
Proportion of population within 30, 60, 90 and 120 minutes travel time of emergency obstetric care in Timor Tengah Selatan district, calculated for travel by ambulance or bus, in the wet or dry season.

**Figure 5.**
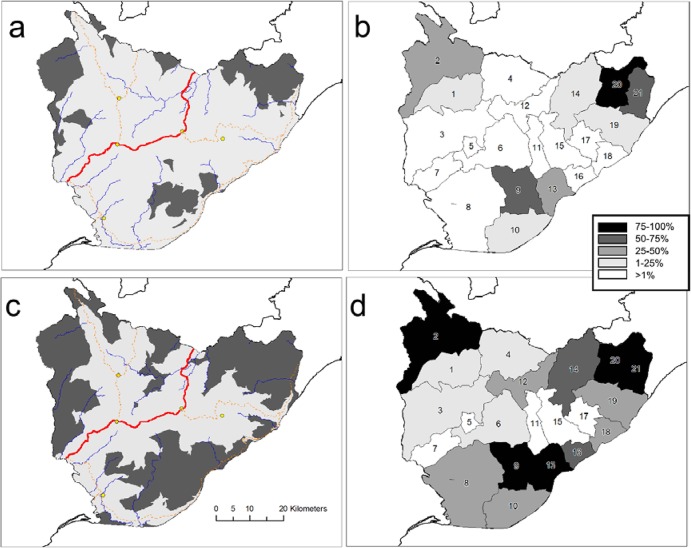
Areas (shaded dark grey) more than two hours travel time by car from emergency obstetric care (EmOC) for the (a) dry season and (c) wet season, and percentage of the population in each sub-district (labeled with numbers) more than two hours away from EmOC during the dry season (b) and during the wet season (d). Location of EmOC facilities are indicated by yellow circles, and national level and district level roads are solid red and dashed orange, respectively.

According to the travel time modelling, a clinic ambulance could reach almost 90% of the population of TTS district within two hours. Even for this best case scenario, over 10,000 women between the age of 15 and 45 years were estimated to be more than two hours from EmOC in the dry season and this number was over 28,000 during the wet season. For the scenario of walking and public transport, about half the population, approximately 50,000 women, were calculated to be remote from EmOC (Figure 4). In the wet season there were five sub-districts in which more than 75% of the population was more than two hours from EmOC compared with one sub-district in the dry season. Women living far from the major roads and in the periphery of the district were remote due to wet season flooding (Figure 5).

**Figure 6.**
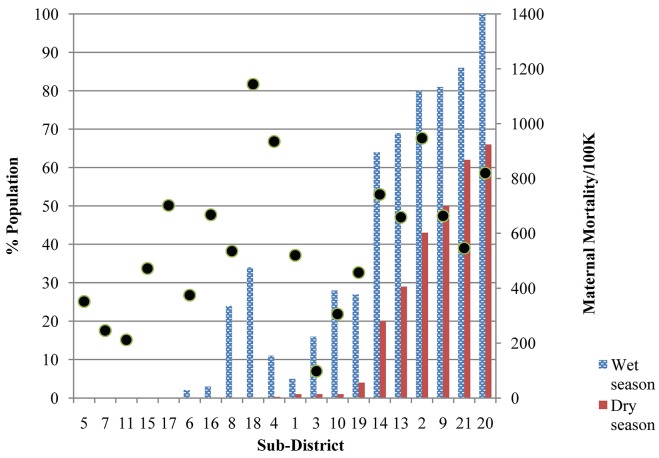
Proportion of population in each district (numbered as in Figure 5) more than two hours away from EmOC by ambulance during the dry season and wet season (solid red and stippled blue, respectively) compared to maternal mortality ration (MMR, deaths /100,000 live births, averaged for 2008–12. The sub-districts are placed (left to right) from the lowest to highest remoteness index, ranked primarily for the dry season and secondly for the wet season.

Generalised linear modelling showed that maternal mortality ratio was positively correlated (*p* < 0.01) with both remoteness index and season; remoteness index (0.37 +/− 0.01) being a stronger predictor than season (0.12 +/− 0.01), however, there was much variation around the fitted relationship (as illustrated in Figure 6). For example, sub-districts 15 and 17 had relatively high maternal death rates despite having relatively low values of the remoteness index (Figure 6). Some districts which were not considered remote in the dry season become isolated during the wet season; for example in sub-districts 12, 16 and 18 the proportion of the population modelled as remote increased from zero in the dry season to over a third in the wet season (Figure 6).

For the sub-district where the ethnographic research was conducted, the maternal mortality ratio was greater than 400 deaths per 100,000 live births and the remoteness index indicated relatively good geographic access to EmOC compared to other sub-districts. Travel time modelling indicated that an ambulance, travelling from an EmOC facility, could reach all the population within two hours in the dry and wet season; and for the travel scenario of walking and bus travel, most of the population was within two hours of EmOC, i.e. 96% and 86%, in the dry and wet seasons respectively. For this sub-district, travel time modeling was refined by using higher resolution data for population distribution, derived from Google Earth. This analysis produced similar results for ambulance travel (i.e. 100% and 98% within two hours of EmOC) but indicated a lower proportion of the population was more than two hours from EmOC for the bus and walking scenario, i.e. 89% and 77%, for the dry and wet seasons respectively.

**Table 5.** The delays in accessing EmOC for eight cases of maternal death in one sub-district in TTS. The stages of greatest delay for each case are indicated by bold text and shaded boxes: in blue for first, green for second delays (defined by Thaddeus and Maine [2]). Note that a skilled birth attendant was only present for case 7.

	Case	First Delay - in deciding to seek care		Second Delay - in reaching care		Season in which death occurred	Modelled travel time for season in which death occurred (min)	
		*Delay in recognising emergency situation*	*Delay in deciding to seek care*	*Delay in transmitting request for help*	*Delay in reaching emergency care*		*Car*	*Walking*
First delay	1 *	**No alarming symptoms were noticed**	Decision to seek care soon after recognising emergency	**Walked to ask traditional birth attendant to come**	Died before help was sent	Dry	47	121
	2	**Emergency situation was not recognised for days**	**Didn't call midwife straight away**	**Walked to get help**	Died before help was sent	Wet	75	156
	3	Emergency situation was recognised	**Did not seek care because travel in wet season too difficult**	No help was sought		Wet	88	161
	4	Family recognised that woman needed care	**Did not seek care because of shame of unmarried pregnancy**	No help was sought		Wet	71	95
	5 *	Need for care was recognised by family and cadre	**Cadre delayed calling midwife until dawn**	Midwife was phoned	Midwife arrived quickly by motor bike	Wet	45	90
Second delay	6 *	Need for care was recognised because of heavy bleeding	Midwife was contacted	**Walked to ask midwife to come**	**Midwife was at another birth**	Dry	38	42
	7	Nurse present at birth recognised emergency because of heavy bleeding after unexpected birth of twins	Decision to request care was made quickly	Travelled by motor bike to clinic by motor bike to request ambulance	**Ambulance was on another call and then broke down on return to clinic**	Wet	80	166
Thirddelay	8	Emergency situation was recognised quickly	Decision to seek help was made quickly	Travelled by motor bike to clinic to request ambulance	Ambulance came and woman was taken to clinic then hospital	Dry	47	121

* indicates cases where death occurred 1–5 hours after the birth. For other cases, death occurred 1–5 days after the birth

## 5. Discussion

The remoteness index, i.e. proportion of population more than two hours modelled travel time from EmOC, was a significant predictor of maternal mortality, particularly when modelled for wet season conditions. However, geographic access was only one of many factors determining remoteness from EmOC [16] and many sub-districts with a low remoteness index had high maternal mortality rates. Ethnographic research [16] of cases of maternal deaths showed that delays in recognising the need for help and deciding to seek help could be fatal. After the emergency had been recognised, the decision to seek care was delayed by perceptions of costs, the performance of ritual customs, stigma associated with unmarried pregnancy and a sense of powerlessness to save the woman (Table 5) [16]. For one case (# 3) perceptions of remoteness was the reason for deciding not to seek care; the family judged the travel time would be too great, requiring walking on tracks in heavy rain and wading through a flooded river. If no decision to seek care was made, travel time became irrelevant. If the decision to seek care was delayed, travel time to request and reach care was even more critical. The remoteness index failed to include many factors that limit access to EmOC and which need to be addressed to improve access to EmOC.

We offer the following ways to improve the modelled estimation of travel time to reach EmOC and to refine the index of remoteness from EmOC: (1) adding the actual sequence of travel involved in reaching care; (2) incorporating a dynamic element to modelling of the factors affecting geographic access; and (3) refining the population distribution data.

### 5.1. Actual travel in reaching care

The actual travel time involved in reaching EmOC was a sequence of travel events to request and reach care. Seeking care can involve several family members, attempting to reach a health worker by foot, one after another, often without success (Table 5) [16]. After delivering the request for care, the total travel time to access care included the time to walk to a village midwife plus the time for the midwife to travel by motor bike to the house of the patient (e.g. cases #1, 2, 6). In other cases, where there was no resident village midwife, travel time comprised time to travel by motor bike to the clinic (cases #7, 8) plus the time for the ambulance to reach the closest road access or the time taken to walk while carrying the patient to that location. If the EmOC required is not available in the ambulance, the time to travel to the clinic also becomes critical. Our ethnographic study investigated only cases of maternal deaths and for most of these cases death occurred before care arrived. Investigation of cases where emergencies occurred but a life was saved (near misses) would give further information about actual travel in the event of a maternal emergency.

Requests for care could be delivered quickly if there was immediate access to a phone. Whilst few villages have functioning public phones, mobile phone ownership is widespread and growing. The coverage of mobile phone reception becomes a key factor for enabling requests for care to be delivered quickly. We propose that isolation resulting from limited coverage of mobile phone signal be incorporated into future GIS modeling of remoteness from health care.

**Figure 7.**
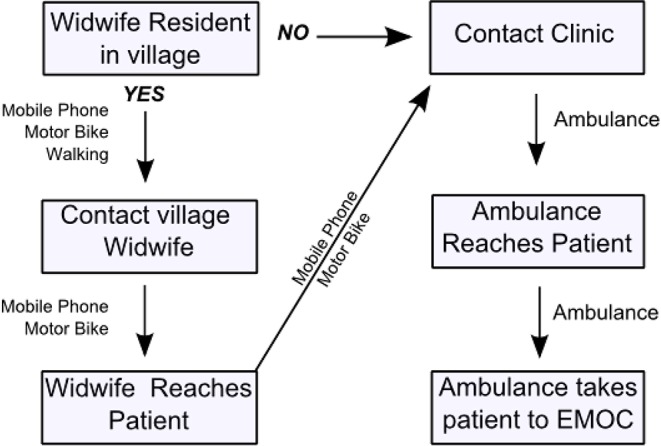
Schematic representation of the sequence of delays in accessing EmOC for cases of maternal death.

In summary, the time taken to reach EmOC is influenced by a sequence of decisions and actions, and delays may occur at any, or several, of the stages in this sequence (Figure 7). A delay in deciding to seek EmOC alone may result in maternal death and so this may occur close to EmOC. When EmOC is sought, actual time to access EmOC is likely to be greater than the modelled travel time which is based on the ideal situation of a request for care being made quickly by phone and an ambulance being sent promptly.

### 5.2. Modelling dynamic factors affecting geographic access

Travel speed is influenced by many variables, such as road condition, which are distributed unevenly through time and space. The static nature of standard forms of modelling travel time, such as used in this research, limits their utility for understanding the highly dynamic nature of these variables. Season was shown to be a significant predictor of maternal mortality ratios, however a simple classification of wet and dry season does not capture the full extent of the variability of seasonal conditions. For example, road damage from flooding and landslides often lasts many months beyond the wet season. Interactive models could incorporate these changing conditions to give more accurate travel time estimates. Interactive models could also be used to consider multiple planning scenarios based on local experience.

Agent based modeling applications are increasingly being used for exploring complexity in infrastructure planning and health behavior modeling [28]. Agent based modelling allows for the simulation of complex systems that integrate GIS data to explore problems of geography, the environment, planning and social systems [29]–[32]. For health access planning the potential exists for agent based modeling to assist local government planners to investigate the likely impacts of changes to continuous variables (such as rainfall or travel speed), and add discrete factors (such as road breaks or new cell phone towers) to predict the impacts on access to services with near real time model outputs.

### 5.3. Refining the population distribution data

The population distribution model derived from the infrastructure mapping from 1996 generally underestimated the number of people remote from health services compared with the higher resolution model produced from recent Google Earth imagery. It is likely that this is because mapping from Google Earth included isolated dwellings which were not included in the coarser infrastructure mapping and these isolated dwellings were more likely to be further from main roads. Although labour intensive, the use of high resolution satellite imagery that is freely available can improve the accuracy of service access modelling.

The combination of spatial and social sciences enriched this study. GIS provided a basis for the strategic selection of sites for ethnographic study and also provided context for interpretation of this social research. This paper illustrates the range of factors influencing access to EmOC however a larger study is required to determine the relative frequency of these geographic, social, financial and cultural factors e.g. [33]–[35]. In this study GIS was used to identify a sub-district with relatively good access to EmOC and relatively high maternal mortality rates. Further social research is planned in sub-districts with a high remoteness index and with relatively low maternal mortality. In addition it would be useful to explore any correlation between geographic remoteness and social-cultural practices related to health care seeking behavior: to investigate whether socio-cultural isolation is correlated with geographical remoteness.

## 6. Conclusion

Whilst travel time was a significant predictor of maternal mortality ratio, ethnographic research has shown that socio-cultural factors can be barriers to requesting care, in these cases obviating considerations of geographic remoteness. Socio-cultural factors contribute to maternal deaths close to EmOC. In addition the modeled travel time did not take into account the complex sequence of actual travel involved in requesting and reaching EmOC revealed by the interviews.

The travel time modeling identified regions that were geographically remote from EmOC where investment in transport infrastructure could improve health outcomes. Improving mobile phone reception has the potential to significantly improve the timeliness of requests for EmOC. The inclusion of mobile phone signal in modeling could inform the strategic positioning of phone towers or signal repeater devices. Planning strategies for the development of infrastructure to improve geographic access to health services could be supported by using agent based modeling coupled with population distribution and dynamic aspects of road access.

This study supports the proposition that integrated research across disciplines is essential for tackling complex problems such as access to health care. In order to define remoteness from services both the physical and cultural barriers need to be understood and addressed.
